# Rejuvenation factor PF4: a potential gatekeeper for neurodegenerative diseases

**DOI:** 10.3389/fnagi.2024.1482922

**Published:** 2024-09-19

**Authors:** Fengju Jia, Xiaoli Shen

**Affiliations:** ^1^School of Nursing, Medical College of Qingdao University, Qingdao, China; ^2^Department of Epidemiology and Health Statistics, Medical College of Qingdao University, Qingdao, China

**Keywords:** PF4, neurodegenerative diseases, aging, neuroinflammation, Parkinson’s disease

## Abstract

Recently, it is discovered PF4 is a cognitive enhancer that improved the cognitive abilities of younger mice and gave older animals their middle-aged acuity back. PF4 works by reducing inflammation during the aging process. As we all known, aging is undoubtedly the main risk factor of neurodegenerative diseases. Furthermore, inflammation has been extensively investigated and attracted even more interest. Therefore, the aim of the proposal is to highlight the worth of PF4 in inflammaging of neurodegenerative diseases, which might provide a potential therapeutic strategy.

An essential aspect of aging is cognitive decline, which is also a common clinical presentation of neurodegenerative diseases (NDDs) such as Parkinson’s and Alzheimer’s disease. The precise etiology of NDDs remains enigma. Aging is undoubtedly the main risk factor. New advances made in understanding the drivers of central nervous system aging May offer an important roadmap to delineating mechanisms of NDDs development. Several major molecular hallmarks of brain aging overlap with mechanisms implicated in neurodegeneration, such as oxidative damage, protein aggregation, and chronic inflammation. Inflammation has been extensively investigated and attracted even more interest as a potential cause of NDDs. “Inflammaging” is dubbed for a persistent low-grade inflammation linked to aging that May be brought on by pro-inflammatory damaged or malfunctioning cells or other heightened innate immune system responses. To precisely address the pathophysiology of NDDs, it is imperative to elucidate the underlying mechanisms of unfavorable circumstances that predispose to neurodegeneration in aging, including the negative influence of neuroinflammation.

Recently, Schroer et al. (2023) report platelet factor 4 (PF4), also called platelet-derived exerkine CXCL4, is a cognitive enhancer that improved the cognitive abilities of younger mice and gave older animals their middle-aged acuity back. The way PF4 works is by reducing inflammation during the aging process and improving cognitive function. The authors initially discover that PF4 is higher in youthful blood plasma. They also observe systemic treatment of exogenous PF4 enhances cognition in old mice, induces molecular alterations associated to synaptic plasticity, and decreases age-related hippocampal neuroinflammation. Meanwhile, Leiter et al. (2023) and Park et al. (2023) respectively highlight the cognitive enhancement role of PF4 in mediating the rejuvenating effects of exercise and longevity factor klotho during physiological brain aging.

Lower PF4 levels is observed in elderly peripheral blood (Weng et al., 2024), nevertheless, the significance of PF4 in NDDs is yet unknown. It is currently rather challenging to determine the molecular mechanistic pathways by which PF4 might exert its anti-aging effects in NDDs. Aging and inflammation are the main pathological changes of NDDs. Now studies have shown that PF4 has anti-inflammatory properties as well as a resist senility (Hemmer et al., 2024). One possible pathway for blood-to-brain communication by which PF4 reverses brain aging is via neuroimmune mechanisms (Lemaitre et al., 2023). Additionally, PF4 has the ability to bind to and activate the thrombopoietin receptor on platelets, which in turn causes platelet aggregation and the activation of the janus kinase (JAK)/signal transducers and activators of transcription (STAT) signaling pathway (Buka et al., 2024). Interestingly, abnormal JAK/STAT signaling pathway activation or phosphorylation has been linked to NDDs such Parkinson’s disease (PD) (Lashgari et al., 2021; Qin et al., 2016). Therefore, it is speculated that JAK/STAT mediated neuroimmune contributes PF4-implicated NDDs.

In order to validate the assessment of PF4 in NDDs and elucidate its exact mechanism, more research is aggressively explored (Figure 1). Consequently, evaluation of the concentration and activity of PF4 in the blood and microglia of patients of NDDs will be fascinating. It is noteworthy to resolve the extent to which PF4 are genuinely responsible for the causal in cognitive decline of NDDs. Furthermore, the exploit of PF4-targeting therapies May be a potential strategy to treat NDDs.

**Figure 1:**
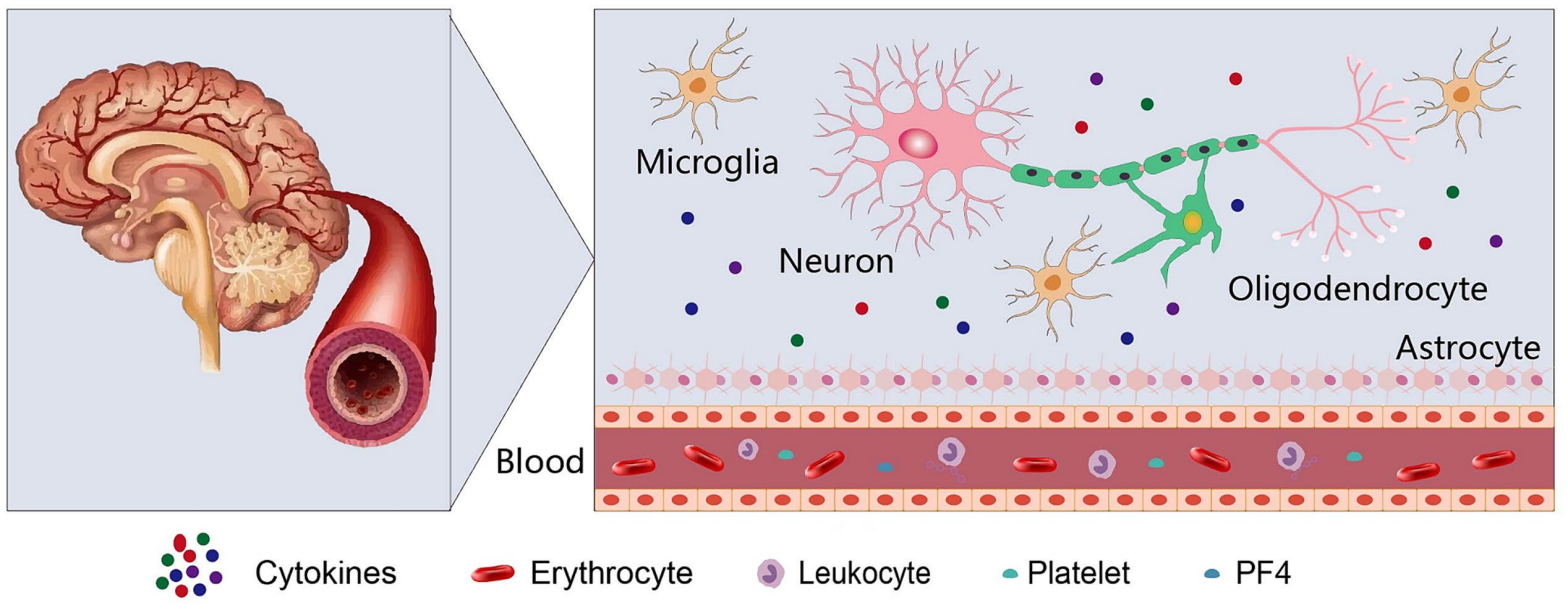
Mode diagram of PF4 in neurodegenerative diseases.

## Data Availability

The original contributions presented in the study are included in the article/supplementary material, further inquiries can be directed to the corresponding author.

## References

[ref1] Buka R. J. Montague S. J. Moran L. A. Martin E. M. Slater A. Watson S. P.. (2024). PF4 activates the c-Mpl-Jak2 pathway in platelets. Blood 143, 64–69. doi: 10.1182/blood.2023020872, PMID: 37883794 PMC10862235

[ref2] Hemmer B. M. Philippi S. M. Castellano J. M. (2024). Youth-associated platelet-derived chemokine reverses brain aging through neuroimmune mechanisms. Trends Mol. Med. 30, 10–12. doi: 10.1016/j.molmed.2023.10.007, PMID: 37945435 PMC10872830

[ref3] Lashgari N. A. Roudsari N. M. Momtaz S. Sathyapalan T. Abdolghaffari A. H. Sahebkar A. (2021). The involvement of JAK/STAT signaling pathway in the treatment of Parkinson's disease. J. Neuroimmunol. 361:577758. doi: 10.1016/j.jneuroim.2021.577758, PMID: 34739911

[ref4] Leiter O. Brici D. Fletcher S. J. Yong X. L. H. Widagdo J. Matigian N.. (2023). Platelet-derived exerkine CXCL4/platelet factor 4 rejuvenates hippocampal neurogenesis and restores cognitive function in aged mice. Nat. Commun. 14:4375. doi: 10.1038/s41467-023-39873-9, PMID: 37587147 PMC10432533

[ref5] Lemaitre P. Tareen S. H. Pasciuto E. Mascali L. Martirosyan A. Callaerts-Vegh Z.. (2023). Molecular and cognitive signatures of ageing partially restored through synthetic delivery of IL2 to the brain. EMBO Mol. Med. 15:e16805. doi: 10.15252/emmm.202216805, PMID: 36975362 PMC10165365

[ref6] Park C. Hahn O. Gupta S. Moreno A. J. Marino F. Kedir B.. (2023). Platelet factors are induced by longevity factor klotho and enhance cognition in young and aging mice. Nat Aging 3, 1067–1078. doi: 10.1038/s43587-023-00468-0, PMID: 37587231 PMC10501899

[ref7] Qin H. Buckley J. A. Li X. Liu Y. Fox T. H. Meares G. P.. (2016). Inhibition of the JAK/STAT pathway protects against α-Synuclein-induced Neuroinflammation and dopaminergic neurodegeneration. J. Neurosci. 36, 5144–5159. doi: 10.1523/JNEUROSCI.4658-15.2016, PMID: 27147665 PMC6123006

[ref8] Schroer A. B. Ventura P. B. Sucharov J. Misra R. Chui M. K. K. Bieri G.. (2023). Platelet factors attenuate inflammation and rescue cognition in ageing. Nature 620, 1071–1079. doi: 10.1038/s41586-023-06436-3, PMID: 37587343 PMC10468395

[ref9] Weng R. Liu J. Yu Q. Yuan H. Qiu Y. Liu H.. (2024). The disparity of platelet factor 4 and platelets in individuals of different ages. Heliyon 10:e34923. doi: 10.1016/j.heliyon.2024.e34923, PMID: 39145023 PMC11320319

